# Data relating to early child development in the Avon Longitudinal Study of Parents and Children (ALSPAC), their relationship with prenatal blood mercury and stratification by fish consumption

**DOI:** 10.1016/j.dib.2016.08.034

**Published:** 2016-08-24

**Authors:** Yasmin Iles-Caven, Jean Golding, Steven Gregory, Alan Emond, Caroline M. Taylor

**Affiliations:** Centre for Child & Adolescent Health, School of Social & Community Medicine, University of Bristol, UK

**Keywords:** ALSPAC, Maternal blood mercury, Child development, Selenium, Fish, Prenatal exposure

## Abstract

As part of the Avon Longitudinal Study of Parents and Children (ALSPAC), measures of early child development were collected using both hands-on expert assessment (on a random 10% sub-sample) by trained psychologists at 18 months using the Griffiths Mental Development Scales (Extended 0–8 years) and from detailed questionnaires completed by the study mothers on the whole cohort using assessments based on the Denver Developmental Screening Test. The development determined by the psychologists on the 10% subsample showed a correlation of 0.49 (R. Wilson, 2003) [9] with the developmental level estimated from the maternal report. Maternal reports were used to determine the associations between prenatal blood mercury levels and scores of social achievement, fine motor skills, gross motor skills and communication at various preschool ages. (For results, please see doi:10.1016/j.neuro.2016.02.006 [1].)

**Specifications table**

**Table:** 

Subject area	Human Biology
More specific subject area	Child development
Type of data	Table
How data were acquired	Longitudinal cohort study questionnaire data; biological assessment
Data format	Edited and analysed
Experimental factors	Maternal self-completion questionnaires and blood assays for mercury
Experimental features	Mean development scores compared with maternal prenatal mercury assays.
Data source location	Former Avon area, centered around Bristol, UK
Data accessibility	Data are within this article.

**Value of the data**

• The ALSPAC dataset contains information on a large number of children in a geographically defined population whose development was monitored over many years.
• The data provide a basis for early identification of adverse effects of environmental exposures (toxicants and other chemicals).
• The data allow detailed analyses of family and social circumstances and their associations with children׳s development.

## 1 Data

In this paper we describe data on child development levels, maternal fish consumption and prenatal blood mercury levels (see Tables and Appendix A Tables).

The ALSPAC study website contains details of all the data that are available through a fully searchable data dictionary: <http://www.bris.ac.uk/alspac/researchers/data-access/data-dictionary/>. Data can be obtained by bona fide researchers after application to the ALSPAC Executive Committee (http://www.bristol.ac.uk/alspac/researchers/access/).

## 2 Experimental design, material and methods

### 2.1 Questionnaire assessments

The ALSPAC design [2] included the distribution of questionnaires by mail to the child׳s main carer for self-completion and return in reply-paid envelopes. At 32 weeks gestation the questionnaire sent to the pregnant mothers included a detailed food frequency questionnaire containing questions on their current consumption of white and of oily fish [3]; women who ate no white or oily fish were defined as non-fish eaters.

Data concerning the child׳s social and communication skills, as well as fine and gross motor development, were asked at 6 months, 18 months, 30 months (2 years 6 months) and 42 months (3 years 6 months). This battery of questions was developed for maternal completion and piloted by ALSPAC from the Denver Developmental Screening Test (DDST) [4]. The battery relates to four different categories: social skills; fine motor skills; communication; and gross motor skills. These questions were adapted to the age of the child and appeared in the maternal self-completion questionnaires at 6, 18, 30 and 42 months (Appendix A Table A1, Table A2, Table A3, Table A4).

The questions at 6 and 18 months concerned whether the study child had reached various milestones, and had the responses (codes):

• Yes does often (2);
• Has only done once or twice (1);
• Has not started yet (0).
  The questions at ages 30 and 42 months had the responses (codes):
• Can do well (2);
• Does this but not very well (1);
• Has not yet done (0).

The scores for each type of skill were summed forming the total development score. The basic details are shown in Table 1 of our companion paper [1]. The Communication items were only asked in the 6 and 18 month questionnaires because features of hearing and speech were asked and tested in much more detail in the later questionnaires. It should also be noted that a consequence of only three skill types being measured at ages 30 and 42 months is that the Total Development scores at these ages are short of this component.

### 2.2 The validation sample

At 18 months, the development of a 10% sample of the study children was assessed by ALSPAC trained psychologists. At the time this assessment was being planned (1993) we were aware that children׳s abilities on the Griffiths Mental Development Extended (0–8 years) scales (GMDS) [5] were improving over time [6], [7], similar to the findings on the Stanford–Binet and other tests of intelligence [8]. We therefore decided to use the extended version of the GMDS so that we would not have a ceiling effect. The normative sample for this extension consisted of 1397 children. The GMDS assesses five areas of development: locomotion, personal/social skills, hearing and speech, hand and eye co-ordination, and performance. The child׳s developmental quotient (DQ) was calculated as the mean of his/her scores on the five subscales.

As shown elsewhere, the child׳s development score obtained using the GMDS estimate of DQ at 18 months was correlated (*r*=0.49) with the score from the questionnaire developmental assessment at that age [9], and that the group of children in the lowest decile of the two scales were related to one another [10].

### 2.3 Mercury measurement

Whole blood samples were collected in acid-washed heparin vacutainers (Becton and Dickinson) by midwives as early as possible in pregnancy. Midwives’ participation in collecting the bloods was voluntary, dependent on time available and was only obtained in two of the three Health Authority areas of the recruitment region for technical reasons. Altogether there were 4484 samples collected at a median gestational age of 11 weeks (range 1–42 weeks mode 10 weeks, interquartile range 9–13 weeks). The social background of the women who gave the samples did not differ from the rest of the ALSPAC population apart from being slightly older and more educated [11]. Samples were stored at 4 °C at the collection site and then sent to the central Bristol laboratory within 0–4 days. These samples were kept at room temperature for up to 3 h during transfer, and were stored at 4 °C as whole blood in the original tubes for 18–19 years before being sent for analysis.

The method of assay of mercury and selenium has been described in detail elsewhere (12). In brief, the laboratory of Robert Jones at the Centers for Disease Control and Prevention (CDC) developed methods to prepare the samples for analysis of whole blood mercury as well as of lead, selenium and cadmium (CDC method 3009.1). Clotted whole blood was digested to remove all clots, before being analyzed using inductively coupled plasma dynamic reaction cell mass spectrometry (ICP-DRC-MS). Two levels of bench quality control (QC) materials as well as a blind QC material were used for daily quality control.

Of the 4484 samples, 4134 were available for mercury and 4287 for selenium assays . All selenium measures were above the level of detection (LOD), but three of the mercury levels were below the LOD of the assay (0.24 μg/L). For these samples, in consideration of the distribution of the mercury levels, a value of 0.7 times the LOD value was considered to be a better estimate of the value than taking a mid-point. The range of mercury levels was from below the LOD to 12.76 μg/L with a median of 1.86 μg/L. For selenium the values ranged from 17.0–324.1 μg/L with a median of 108.0 μg/L).

### 2.4 Publications

Publications using the questionnaire measurements of child development are shown in Table 1. Beneficial associations were found for prenatal fish intake [13], [14], prone sleeping [15] and parenting behaviours [16], and negative associations with maternal prenatal depression [17]. In regard to prenatal fish consumption using the food frequency questionnaire, Golding et al. [12] showed that there were strong associations of child development with various dietary constituents including fish intake.

### 2.5 Associations with prenatal mercury

In our parallel paper [1] we show there were no negative associations of prenatal blood mercury with the total development scores after adjustment using the continuous scales of each measurement. Here we show the adjusted results for the individual components of the development measures (Table 2, Table 3). All the results showing *P*<0.10 indicate that at the level of prenatal blood mercury in this study there were no adverse associations.

## Funders

The UK Medical Research Council and the Wellcome Trust, United Kingdom **(**Grant ref: 102215/2/13/2**)** and the University of Bristol, United Kingdom currently provide core support for ALSPAC. The assays of the maternal blood samples were carried out at the Centers for Disease Control and Prevention with funding from NOAA, and the statistical analyses were carried out in Bristol with funding from NOAA and support from the Intramural Research Program of NIAAA, NIH. CMT was supported by a Wellcome Trust Career Re-Entry Fellowship (Grant ref: 104077/Z/14/Z). The funders had no involvement in the study design nor in the collection, analysis and interpretation of the data.

## Competing financial interests

The authors have no competing interests.

## Figures and Tables

**Table A1:** Items relating to social skills.

	**Milestones**	**AGE**			
		**6 m**	**18 m**	**30 m**	**42 m**
1	Looks at older people faces	+	–	–	–
2	Smiles when you smile at him/her	+	–	–	–
3	Does not want to let go of a toy when it is being taken away	+	–	–	–
4	Can feed him/herself with a rusk or similar	+	–	–	–
5	Tries to get to a toy that is out of reach	+	–	–	–
6	Is shy when he first meets a stranger	+	+	–	–
7	Plays peek-a-boo	+	+	–	–
8	Plays pat-a-cake	+	+	–	–
9	Is able to drink from a cup	+	+	+	–
	Is able to drink from a cup without spilling it	–	–	–	+
10	Indicates what he/she wants without crying for it	+	+	+	–
	Asks for what he/she wants without crying for it	–	–	–	+
11	Copies me doing the housework	–	+	+	+
12	Uses a spoon without spilling much	–	+	–	–
13	Helps in the house with simple tasks	–	+	+	+
14	Can take off clothes with help	–	+	+	+
15	Can put shoes on (without fastening them)	–	+	+	+
16	Can wash and dry hands	–	+	+	+
17	Lets me know when wants to go to the lavatory	–	+	–	–
18	Will play happily on own	–	+	–	–
19	Eats with a spoon and/or fork	–	+	+	+
20	Can put on a T-shirt by him/herself	–	–	+	+
21	Can brush teeth (with help)	–	–	+	+
22	Can get dressed without help	–	–	+	+
23	Plays card games or board games	–	–	+	+
24	Prepares breakfast cereal to eat	–	–	+	+

Key: + item in the questionnaire; − item not in the questionnaire.

**Table A2:** Items relating to fine motor coordination.

	**Milestones**	**AGE**			
		**6 m**	**18 m**	**30 m**	**42 m**
1	Puts hands together	+	–	–	–
2	Can hold a rattle	+	+	–	–
3	Can focus eyes on a small object such as a raisin	+	+	–	–
4	Reaches for objects	+	–	–	–
5	In a sitting position looks about for a hidden object	+	–	–	–
6	In a sitting position can pick up 2 objects at once	+	–	–	–
7	He can pick up a small object such as a raisin	+	+	–	–
8	Can pass an object from one hand to another	+	+	–	–
9	Can bang together two similar objects that he is holding	+	+	+	–
10	Grabs objects using the whole hand	+	+	+	+
11	Can pick up a small object using forefinger and thumb only	+	+	+	+
12	Will use a pencil and scribble	–	+	+	+
13	Can build a tower putting one object on top of another	–	+	+	+
14	Can build a tower of 3 bricks	–	+	–	–
15	Can build a tower of 4 bricks	–	+	+	+
16	Can build a tower of 8 bricks	–	+	+	+
17	Can copy a vertical line with a pencil	–	+	+	+
18	Points to what he/she wants	–	+	–	–
19	Will turn the pages of a book	–	+	+	+
20	Can wiggle his/her thumb	–	–	+	+
21	Can copy a circle and draw it more or less	–	–	+	–
	Can draw a circle	–	–	–	+
22	Can build a tower of 6 bricks	–	–	+	+
23	Can fit shapes in a board	–	–	+	+
24	Can thread beads on a string	–	–	+	+
25	Can use his/her right hand to draw	–	–	+	+
26	Can use his/her left hand to draw	–	–	+	+
27	Can copy a plus sign and draw it more or less	–	–	–	+
28	Can copy a square and draw it more or less	–	–	–	+
29	Can undo big buttons	–	–	–	+
30	Can fasten big buttons	–	–	–	+

Key: + item in the questionnaire; − item not in the questionnaire.

**Table A3:** Items relating to communication skills.

	**Milestones**	**AGE**	
		**6 m**	**18 m**
1	When a bell rings he/she moves or makes a noise	+	+
2	Makes noises other than crying	+	–
3	Laughs	+	–
4	Squeals	+	–
5	Turns toward someone when they are speaking	+	+
6	Tries to copy what you say	+	+
7	Says ‘dada’ and ‘mama’	+	–
8	Says ‘dada’ and ‘mama’ and knows what they mean	+	+
9	Says at least 3 other words and knows what they mean	+	+
10	Combines 2 different words (e.g. nice dinner)	–	+
11	Can point to his/her toes when asked	–	+
12	Uses plurals (e.g. cats, toys)	–	+
13	Gives his/her first name	–	+
14	Gives his/her first and last name	–	+
15	Understands the word ‘cold’	–	+
16	Understands ‘hungry’	–	+
17	Can name 3 colours, even if he/she doesn’t get them right	–	+
18	Makes negative statements (e.g. no bath)	–	+

Key: + item in the questionnaire; - item not in the questionnaire

**Table A4:** Items relating to gross motor coordination.

	**Milestones**	**AGE**			
		**6 m**	**18 m**	**30 m**	**42 m**
1	In a sitting position can keep his head steady	+	–	–	–
2	Lying on his stomach can lift his chest and shoulders while supporting them with his/her arms	+	–	–	–
3	Can roll over	+	–	–	–
4	Is able to bear some weight on his/her legs when held in a standing position	+	–	–	–
5	Can sit up without being supported	+	–	–	–
6	Can stand up while holding on to something such as furniture	+	–	–	–
7	Can pull himself up to a standing position by holding onto a piece of furniture	+	–	–	–
8	Can get from a standing position to a sitting position	+	–	–	–
9	Can walk holding onto a piece of furniture	+	–	–	–
10	While lying on his stomach can lift his head	+	–	–	–
11	Can stand up for a moment without any support	+	+	–	–
12	From a standing position can stoop and return to standing	+	+	+	+
13	Can crawl on hands and knees	+	–	–	–
14	Can stand alone for at least a minute without holding on to anything	–	+	–	–
15	Can walk while holding someone׳s hand	–	+	–	–
16	Can walk alone for at least 5 steps	–	+	–	–
17	Can move around while shuffling on his/her bottom	–	+	–	–
18	Can kick a ball	–	+	+	+
19	Can throw a ball	–	+	+	+
20	Can balance on one foot for at least 1 second	–	+	+	–
	Can balance on one foot for at least 4 seconds	–	–	–	+
21	Can jump up and down	–	+	+	+
22	Can climb stairs	–	+	–	–
23	Can walk	–	–	+	+
24	Can walk backwards 5 steps	–	–	+	+
25	Runs	–	–	+	+
26	Can walk up steps	–	–	+	–
	Can walk up steps – one foot on each step	–	–	–	+
27	Can hop	–	–	+	–
	Can hop at least twice on one foot	–	–	–	+
	Can hop for at least 5 feet	–	–	–	+
28	Can walk on tiptoe	–	–	+	–
	Can walk on tiptoe for at least 9 feet	–	–	–	+
29	Can stop from a full run within 2 steps	–	–	–	+
30	Can walk down steps like an adult – one foot on each step	–	–	–	+
31	Can jump over an obstacle (e.g. toys on floor)	–	–	–	+

Key: + item in the questionnaire; − item not in the questionnaire.

**Table 1:** Publications using measures of child development in ALSPAC as outcomes of environmental exposures.

**Authors**	**Outcomes**	**Environment**	**Results**
Deave et al. 2008 [17]	18 m	Maternal depression	Prenatal but not postnatal depression was associated with reduced score.
Hibbeln et al. 2007 [13]	6-42 m	Prenatal fish intake	Higher fish consumption was associated with better performance on 6/14 sub-categories.
Daniels et al. 2004 [14]	18 m	Prenatal fish and mercury in umbilical cord	No association with mercury; positive association with fish.
Chittelborough et al. 2011 [18]	18 m	Teenage mother	No associations of maternal age with child׳s development
Dewey et al. 1998 [15]	6 m; 18 m	Sleeping position	Prone sleeping associated with advanced development at 6 m but not at 18 m.
Gutman & Feinstein 2010 [16]	6,18,30,42 m	Parenting	Strong beneficial effects

**Table 2:** Association of prenatal mercury exposure/fish consumption with child development. A positive beta indicates better performance. Association (change in points of development score for each SD of mercury) between prenatal mercury exposure and components of child development scores after adjustment for age at assessment and sex of child, maternal age, parity, education, smoking, alcohol, housing tenure, household crowding, family adversity score, life events in the first half of pregnancy and whether the child was breast fed. Results with *P*<0.100 are shown in bold.

	***N***	**β [95% CI]**	***P***
**Development scores at 6 months:**			
Social skills			
All children	2721	**+0.267 [+0.104,+0.429]**	**0.001**
Mother did not eat fish	354	+0.145 [−0.516, +0.806]	0.666
Mother ate fish	2354	**+0.278 [+0.106,+0.451]**	**0.002**
Fine motor			
All children	2723	**+0.192 [−0.017, +0.401]**	**0.072**
Mother did not eat fish	354	+0.690 [−0.138, +1.519]	0.102
Mother ate fish	2356	+0.182 [−0.041, +0.404]	0.110
Communication			
All children	2723	+0.072 [−0.026, +0.170]	0.150
Mother did not eat fish	354	−0.281 [−0.692, +0.130]	0.180
Mother ate fish	2356	+0.069 [−0.034, +0.173]	0.191
Gross motor			
All children	2730	+0.004 [−0.167, +0.176]	0.959
Mother did not eat fish	355	−0.300 [−1.035, +0.435]	0.422
Mother ate fish	2361	+0.072 [−0.107, +0.251]	0.430
**Development scores at 18 months:**			
Social achievement score			
All children	2649	+0.113 [−0.022, +0.248]	0.102
Mother did not eat fish	337	+0.194 [−0.294, +0.682]	0.434
Mother ate fish	2300	+0.078 [−0.067, +0.223]	0.293
Fine motor score			
All children	2649	**+0.113 [+0.004,+0.222]**	**0.043**
Mother did not eat fish	337	**+0.431 [+0.037,+0.826]**	**0.032**
Mother ate fish	2300	+0.090 [−0.027, +0.207]	0.132
Communication score			
All children	2650	**+0.194 [+0.026,+0.362]**	**0.024**
Mother did not eat fish	338	+0.192 [−0.413, +0.797]	0.533
Mother ate fish	2300	**+0.160 [−0.020, +0.351]**	**0.081**
Gross motor score			
All children	2644	+0.043 [−0.061, +0.147]	0.417
Mother did not eat fish	338	+0.152 [−0.223, +0.526]	0.426
Mother ate fish	2294	+0.016 [−0.095, +0.127]	0.777
**Development scores at 30 months:**			
Social achievement score			
All children	2457	+0.079 [−0.056, +0.214]	0.250
Mother did not eat fish	318	+0.289 [−0.182, +0.760]	0.228
Mother ate fish	2128	+0.030 [−0.115, +0.176]	0.681
Fine motor score			
All children	2464	+0.047 [−0.094, +0.189]	0.511
Mother did not eat fish	320	+0.146 [−0.363, +0.654]	0.574
Mother ate fish	2133	+0.024 [−0.128, +0.176]	0.754
Gross motor score			
All children	2461	+0.030 [−0.068, +0.127]	0.550
Mother did not eat fish	319	+0.185 [−0.159, +0.529]	0.291
Mother ate fish	2131	+0.023 [−0.082, +0.127]	0.670
**Development scores at 42 months:**			
Social development score			
All children	2394	**+0.156 [+0.036, +0.276]**	**0.011**
Mother did not eat fish	311	+0.175 [−0.295, +0.644]	0.465
Mother ate fish	2073	**+0.149 [+0.020, +0.277]**	**0.023**
Fine motor score			
All children	2397	+0.122 [−0.030, +0.273]	0.115
Mother did not eat fish	312	+0.118 [−0.506, +0.742]	0.710
Mother ate fish	2075	+0.091 [−0.070, +0.252]	0.269
Gross motor score			
All children	2401	+0.105 [−0.038, +0.249]	0.149
Mother did not eat fish	314	**+0.807 [+0.235, +1.379]**	**0.006**
Mother ate fish	2077	+0.028 [−0.124, +0.180]	0.717

**Table 3:** Associations (change in points of development score for each SD of mercury) between prenatal mercury exposure and child development score after adjustment for age at assessment and sex of child, maternal age, parity, education, smoking, alcohol, housing tenure, household crowding, family adversity score, life events in the first half of pregnancy and whether the child was breast fed. The analyses are presented for all offspring as well as for the two subgroups concerning whether or not the mother ate fish prenatally. Results with *P*<0.100 are shown in bold.

	***N***	**β [95% CI]**	***P***
**Development scores at 6 months:**			
Social skills			
All children	2721	**+0.247 [+0.079,+0.415]**	**0.004**
Mother did not eat fish	354	+0.136 [−0.186, +0.468]	0.688
Mother ate fish	2354	**+0.260 [+0.081,+0.438]**	**0.004**
Fine motor			
All children	2723	**+0.184 [−0.032, +0.400]**	**0.094**
Mother did not eat fish	354	**+0.708 [−0.125, +1.541]**	**0.095**
Mother ate fish	2356	+0.166 [−0.064, +0.396]	0.158
Communication			
All children	2723	**+0.089 [−0.012, +0.191]**	**0.085**
Mother didn’t eat fish	354	−0.278 [−0.692, +0.136]	0.187
Mother ate fish	2356	+0.089 [−0.018, +0.196]	0.104
Gross motor			
All children	2730	−0.018 [−0.195, +0.159]	0.845
Mother did not eat fish	355	−0.286 [−1.025, +0.452]	0.446
Mother ate fish	2361	+0.040 [−0.145, +0.225]	0.670
**Development scores at 18 months:**			
Social achievement score			
All children	2649	**+0.121 [−0.019, +0.261]**	**0.090**
Mother did not eat fish	337	+0.199 [−0.290, +0.689]	0.423
Mother ate fish	2300	+0.087 [−0.063, +0.238]	0.255
Fine motor score			
All children	2649	**+0.131 [+0.018,+0.244]**	**0.023**
Mother didn’t eat fish	337	**+0.419 [+0.024,+0.814]**	**0.038**
Mother ate fish	2300	**+0.119 [−0.003, +0.240]**	**0.055**
Communication score			
All children	2650	**+0.170 [−0.003, +0.344]**	**0.054**
Mother did not eat fish	338	+0.183 [−0.423, +0.790]	0.553
Mother ate fish	2300	+0.139 [−0.047, +0.326]	0.144
Gross motor score			
All children	2644	+0.064 [−0.044, +0.171]	0.246
Mother did not eat fish	338	+0.146 [−0.229, +0.522]	0.444
Mother ate fish	2294	+0.046 [−0.069, +0.160]	0.432
**Development scores at 30 months:**			
Social achievement score			
All children	2457	+0.105 [−0.035, +0.246]	0.142
Mother did not eat fish	318	+0.305 [−0.168, +0.778]	0.205
Mother ate fish	2128	+0.055 [−0.097, +0.206]	0.481
Fine motor score			
All children	2464	+0.083 [−0.064, +0.230]	0.267
Mother did not eat fish	320	+0.141 [−0.371, +0.652]	0.589
Mother ate fish	2133	+0.071 [−0.088, +0.230]	0.379
Gross motor score			
All children	2461	+0.049 [−0.052, +0.150]	0.342
Mother did not eat fish	319	+0.162 [−0.182, +0.507]	0.355
Mother ate fish	2131	+0.056 [−0.053, +0.165]	0.318
**Development scores at 42 months:**			
Social development score			
All children	2394	**+0.180 [+0.056,+0.304]**	**0.004**
Mother did not eat fish	311	+0.205 [−0.269, +0.678]	0.396
Mother ate fish	2073	**+0.170 [+0.037,+0.303]**	**0.012**
Fine motor score			
All children	2397	**+0.136 [−0.020, +0.293]**	**0.088**
Mother did not eat fish	312	+0.139 [−0.491, +0.769]	0.664
Mother ate fish	2075	+0.106 [−0.060, +0.273]	0.210
Gross motor score			
All children	2401	+0.119 [−0.028, +0.267]	0.113
Mother did not eat fish	314	**+0.843 [+0.267,+1.419]**	**0.004**
Mother ate fish	2077	+0.037 [−0.121, +0.195]	0.645

## Appendix A The items in the questionnaire assessments

See Appendix Table A1, Table A2, Table A3, Table A4.
